# A Land of Charity

**Published:** 1889-02-02

**Authors:** 


### A Land of Charity.

By a Colonist.

If there is one thing more than another that a colonist who returns to England notices, it is the vast system of charity which prevails at home. He walks by a huge hospital, with walls and columns towering into the air, until the roof is hidden in smoke and mist, and he reads the announcement, "Supported by voluntary contributions." Every newspaper he picks up contains some reference to the wills and bequests of departed philanthropists, who have left portions of their worldly goods, now no longer needed by them, to institutions of a charitable nature. And if he chooses to make a few inquiries, or read the pamphlets which these institutions publish, he will find column upon column of acknowledgments, and receipts to the value of thousands—tens of thousands of pounds. The conclusion which his mind arrives at is that England is a land of charity: there is no alternative. And, as I have remarked, this is, to the thinking traveller, perhaps the most noticeable feature of the motherland.

In those young countries which are striving to establish themselves in the world of states, charity in this form is little known. There are hospitals, it is true, but they could almost be reckoned on the fingers. Benevolence, no doubt, exists, but it consists rather of private acts and little kindnesses done by man to man. A family deprived suddenly of its breadwinner looks for assistance to those immediately surrounding it, and is duly provided for. It is not thrown upon the rates or driven to seek relief from a particular organisation founded for the purpose. And this is because (1), there is a scarcity of population, and (2), less real distress. The multiplicity of souls in London is so huge that an individual is sunk in the ocean of humanity which surrounds him. He may plead for a crust in the streets and be passed by thousands, who neither heed his cry or answer it. He may wander past a dozen homes or asylums and yet not be able to establish his claim for admittance in one of them. A little too sick for some, and not ill enough for others, he may enter upon the last long journey while trying to accomplish one of a merely mundane nature. All this is obvious enough. Vast as are the systems of relief, and immense though the sums may be that are yearly set apart for charity, they can never be quite sufficient it must be feared. Still they achieve very great results, and the land has a distinct claim to be called a land of charity.

The Australias have their poor and their sick, and it may be that the aid of the good Samaritan will some day be fully needed; but at present this is not the case. The air is less polluted; the crowding is not so intense. Houses may be humble, but they have sufficient rooms for families to live in without conditions of health and propriety being violated. The consequence is that fewer beings are brought into the world as mere receptacles in which disease—hereditary and imported—is concentrated. The lungs breathe oxygen, amongst other things; the limbs are developed, and strong; and, most important of all, the minds are buoyant, ambitious, and capable of receiving good impressions. These are conditions which nautrally mark a young country; and their existence lessens the need for charity.

Another feature must be noticed. Colonies do not possess the wealth that abounds in older lands; and therefore they could not endow such numerous institutions, even were the latter needed. It would be interesting to add up the amount of money, the millions that have been spent in England, in building and maintaining hospitals, homes, and similar concerns. Although it is vague work to guess at what the probable total would be, it is likely enough that it would equal or exceed the aggregate of many of the colonial debts. Money must be plentiful enough in a country when its capitalists are content to enjoy the sweet security of the "Goschens," and draw interest at a rate that is rapidly approaching the vanishing point. Many colonists have great wealth, and most are comfortably off, but young lands have none of the accumulated capital, the hoarded millions which are found in the older ones. Perhaps when they come to have their stores of wealth, they will also have their poor, and sick, and orphans. Let us hope, in such an unhappy event, they will erect as many monuments to that beautiful trait of human nature, charity and sympathy, as England has done.

During the decade of years which I have spent in the south, I do not remember having seen a single beggar. And if there are any there, it is only in the very largest centres. In New Zealand a particular proportion of the general rates is set apart for distribution by the County Councils among the widows, orphans, or disabled of their several districts. And there is no such thing as a workhouse. But relief is almost always temporary, and very often not really required. As an example, the case of a widow may be mentioned, who had been receiving something weekly for a considerable time. It was found on inquiry that the good lady had forty acres of good land (freehold), nine cows, a cottage, furniture, and many implements, etc., on the farm. There can, indeed, be no real distress when food may be had of a neighbour for the asking, and a hut for the building.

Neither do you come across those terrible deformities which are so common in this country. No doubt the unhealthy conditions which surround the poor here are responsible for the wretched specimens of humanity which we meet with daily in the streets. The colonies do not seem to have their blind, or maimed, or halt! But, having them, it must be said for England that every effort is made to alleviate suffering and relieve distress. And even what may be called promiscous charity—*i.e.*, that which is given haphazard in the streets—assumes large proportions. When I went abroad, ten years ago, one of the last pennies I gave away was to the blind man who sits and nets in the lane adjourning St. Martin's-in-the-Fields. On my return, after roaming the wide world o'er, I happened to walk past the spot and there, to my astonishment, sat the old gentleman, just as though he had never moved since I had left! I could hardly believe my eyes; it was like Rip van Winkle waking up from sleep. The dog was a new comer though. His predecessor had lived his life away (a faithful companion to a superior being, and a valued one, I have no doubt) picking up the pennies as they dropped, and running with them to the poor blind master's open hand with as much glee as if he thoroughly understood that another loaf could be bought, or another bone. But charity is evidently plentiful enough, for our blind friend is fatter than he was ten years ago, and looks even younger.

Yes, though it contains many poor, many sick, many sad and sorry, England is a land of charity. That is its great, distinguishing, its crowning feature.

